# Framework for assessing bridges construction impact on work zone traffic using BrIM

**DOI:** 10.1038/s41598-023-50404-w

**Published:** 2024-01-02

**Authors:** Mohamed Marzouk, Ahmed Elsayed

**Affiliations:** 1 Structural Engineering Department, Faculty of Engineering, Cairo University, Giza, 12613 Egypt; 2 Integrated Engineering Design Management Program, Faculty of Engineering, Cairo University, Giza, Egypt

**Keywords:** Civil engineering, Information technology

## Abstract

This research presents a framework for visualizing bridge construction impact on work zone traffic using Bridge Information Modelling (BrIM) and Google Maps API. The framework consists of four modules. The first module contains the construction data of the bridge by reporting the construction's daily progress. The second module is designated to model traffic data of the work zone using Google Maps API, traffic Survey counting, and Google Earth Images. The third module performs a traffic simulation for the work zone, and calculating the user cost of different stages. The fourth module visualizes the bridge's construction using Bridge Information Modelling (BrIM) and virtual reality in the Twinmotion engine to demonstrate the construction stages and the corresponding traffic states. An actual case study of El-Nahas Bridge in Cairo city-Egypt is presented to demonstrate the main features of the developed framework and its practical aspects. The case study results reveal that the impact of the construction stages varies on work zone traffic throughout the project period. The impact greatly influences the traffic status at the beginning of the project associated with high user costs. Then, the impact declines in later stages depending on the type of construction activities in each stage.

## Introduction

Bridges facilitate the transportation of traffic volumes from the origin to the destination inside cities. Construction of bridges follows many different construction methods depending on the project's nature. It occupies spaces of the surrounding environment called work zones, where the construction operations occur. Work zones affect the surrounding environment in general and the traffic flow specifically. The effects of the bridge construction work zones extend to many aspects of the surrounding environment. The traffic status of the work zone is one of the most critical aspects that is deeply affected by these construction activities. Harb^1^ developed a statistical analysis indicating that in the case of single-vehicle crashes, trucks, and large trucks are 44.6% more likely to be involved in work zone crashes than nonwork zones. Also, motor vehicles are 23.5% more prone to crashes in a work zone with poor or no lighting in the dark than in the same conditions in nonwork zones. Work zones have become the second largest contributor to the nonrecurring delay of US highways. This caused nearly 24% of all nonrecurring and 10% of overall delays, as illustrated in the report prepared by the Federal highway Institute in Texas and issued by Cambridge Systematics^2^. Analysis done by Raju et al.^3^ showed that due to construction activities, the drivers are found to be more in conservative mode; as a result, the stream speeds drop from 70 to 50 km/h, whereas the capacity values in the construction work zone were reduced by about 10%. Also, studies showed that three lanes capacity in one direction is found to be declined by 30% in the construction zone. Saha and Sisiopiku^4^ demonstrated that vehicles could drive at regular speed during the uncongested situation, but speeds might decline by 31.6–56.1% of the regular speed in work zones. Accordingly, new strategies are needed to mitigate the effect of bridge construction activities in the work zone on the traffic network.

The main objective of this study is to develop a framework that optimizes the construction cost of the bridges in the work zone by providing visual information for the stakeholder of the bridges during the construction stages. To fulfill the main objective of the study, the following sub-objectives are achieved; Providing the required data that effectively describes the changeable status of the work zone activities, including construction data and traffic data. Developing a real-time web-based platform that relates to Google maps API to reflect the work zone traffic changes during the bridge construction. Analyzing the data collected using traffic simulation, user cost calculations, and time–cost trade-off to determine the optimum work zone alternative detours and project duration. Visualizing the construction stages and the work zone traffic status through BIM and Virtual reality (VR) technology to demonstrate the expected scenarios in the work zone.

## Literature review

Different agencies made efforts to adopt BIM for bridges and structures. Transportation Pooled Fund (TPF)^5^ is a program provided by the Federal Highway Administration (FHWA)^6^. TPF-5(372), a project of the TPF program, aimed to establish a standard for semantic and geometric information of bridges that is common in the United States. The outcomes of the project (TPF-5(372)) are the Development of an Information Delivery Manual (IDM), the Creation of a Model View Definition (MVD), the Development of Software Certification Materials, and the Deployment of Stakeholders Training. BIM also enables assessing the different scenarios using visual qualitative and quantitative information and helps choose the best scenario with the minimum time and cost. Marzouk and Hisham^7^ presented a Bridge Information Modeling (BrIM) framework which used Structured Query Language (SQL) commands to visualize bridge components based on data from the database and inspection spreadsheets. Besides, the authors proposed a way for performing precise cost estimates utilizing BrIM as a supporting tool. Adibfar et al.^8^ created the digital twin of the bridge by integrating real-time Weigh-in-motion (WIM) data via visual scripting into a BrIM model. By using this method, the digital twin of the bridge might feed weight sensor data, increasing the amount of data that is utilized by various stakeholders. Through proactive planning and improved use of the available data, the study's findings will contribute to the sustainability, preservation, and resilience of bridges. Jeon et al.^9^ implemented a data-driven modeling approach using basic bridge information and integrated an inventory code system to effectively manage and utilize the data. Additionally, mapping and deep learning-based vectorization were taken into consideration for managing inspection information, and features for bridge assessment, dashboards, and reporting were incorporated to support decision-making. A variety of stakeholders, including bridge owners, managers, and site inspectors, provided information and functional requirements for the study.

Akanbi et al.^10^ proposed a framework in the architecture, engineering, and construction (AEC) sector for automatically: (1) processing existing 2D bridge drawings; (2) converting these record drawings into three-dimensional (3D) information models; and (3) converting 3D information models into industry foundation class (IFC) files. The state-of-the-art method was used to compare the created 3D models using the suggested framework against other developed 3D models. The framework that has been built can be utilized to create semiautomated algorithms that create 3D models and IFC output files from bridge drawings in the portable document format (PDF). This is demonstrated by the results of experiments. The created models are of comparable quality and the suggested method generates them in 3.33% of the time required by the state-of-the-art method.

Rohani et al.^11^ used four-dimensional computer-aided design models to build visual simulation management tools for infrastructure projects such as highways and bridges. Jeong et al.^12^ described a data management architecture for bridge monitoring applications. NoSQL database systems like MongoDB and Apache Cassandra were used to manage the unstructured bridge information model data. Managing the traffic in the work zone during construction is essential to reduce traffic delays and preserve workers' and motorists' safety. Effective work zone traffic management includes evaluating the work zone's impacts and recording the strategies needed for mitigating these impacts in a transportation management plan (TMP). Many studies have been conducted to optimize the work zone length and schedule in the last years. Zhao et al.^13^ optimized work-zone schedules and associated characteristics (e.g., maintenance crew, work-zone lengths, and diversion rates) to achieve the minimum total cost (i.e., road user cost and agency cost) by implementing effective traffic management strategies (i.e., traffic diversion or shoulder use). Genetic algorithms (GA) technique was developed using MATLAB to find the optimal work-zone schedule and associated maintenance crew that minimizes the total cost.

Marzouk and Fouad^14^ presented a framework for determining the optimal length of a highway resurfacing work zone. The framework could calculate the total duration and cost of resurfacing by using simulation analysis to model highway resurfacing operations while accounting for associated uncertainties. Singh et al.^15^ modeled work zones using the cellular automata model to study the effects of work zones on traffic flow. Yu et al.^16^ studied three main factors, namely, (1) the work zone maintenance time window, (2) network road safety and work team workload, and (3) innovatively introduced speed limit control strategies. The study's goal was to reduce traffic flow speed variation and thus improve traffic safety for the entire road segment. Memarian et al.^17^ developed a traffic diversion model to recommend the best alternate route for drivers during construction activities. The developed models and algorithms evaluated a potential diversion route to optimize network performance while considering the drivers' behaviors in following the proposed alternate route during a closure. Lethanh et al.^18^ developed a GIS-based program in their research to discover optimal intervention programs for large infrastructure networks using a linear optimization model that can be directly linked to a GIS. Zhou et al.^19^ proposed a numerical approach for evaluating the structural safety of widened bridges undergoing maintenance work that requires a partial lane closure on the new bridge and traffic control measures to assure bridge safety.

The purpose of work zone traffic control is also used to provide a safe work zone for workers within the roadway while facilitating the safe and orderly traffic flow of all road users. FHWA^20^ proposed a traffic control approach to facilitate traffic flow safely through and around the work zones. The approaches included three main sections: (1) control strategies, (2) traffic devices, (3) project coordination strategies, and innovative construction strategies. Saha and Sisiopiku^4^ investigated the operational impacts of two Temporary Traffic Control (TTC) strategies, static late and early merge control, with 3-to-1 lane-drop configurations. They considered a hypothetical work zone on I-65 in Birmingham, AL using VISSIM software for simulation modeling. Based on the findings from this investigation, it is recommended that long-term work zones with 3-to-1 lane closures should be avoided. Instead, short-duration closures should be considered, preferably during non-peak periods, to minimize the impacts on traffic operations. Malveaux et al.^21^ conducted preliminary research and proof-of-concept development work to use unmanned aerial vehicles (UAVs) in real-time traffic monitoring of highway construction zones to generate real-time alerts for motorists, construction workers, and first responders.

The time–cost trade-off is considered to be one of the fundamental problems in project management. It aims at minimizing the project duration with the least expenditure of resources. Elmenshawy and Marzouk^22^ developed a model to automatically generate a schedule using BIM and propose optimum solutions for the time–cost trade-off problem (TCTP) resulting from the different scenarios offered to the customer. Agdas et al.^23^ illustrated that the metaheuristic approach effectively solved large-scale TCT construction problems. The study demonstrated that as networks of up to 630 variables were solved using a developed genetic algorithm (GA) with an accuracy of (> 3% deviation) in less than 10 min, networks of up to 6300 variables could be solved by the same method with an accuracy of (> 7% deviation) but of longer processing time. Bettemir and Talat^24^ proposed the optimum or the near optimum solution for the TCTP by applying the minimum cost slope based on the network analysis algorithm. The elimination algorithm decreased the feasible crashing options produced in large projects. Biswasa et al.^25^ used a critical path and a heuristic method to determine the crash duration and crash costs. A regression analysis was performed to identify the relationship between time and cost to formalize an optimization problem model. Tedla^26^ aimed at optimizing the time and the profit of the construction highways projects taking into consideration cash flow analysis, the future cost of each activity, and the available credit to the individual objectives.

From these reviews, some research gaps and challenges are required to be covered in the proposed approach. These gaps include the availability of real-time traffic data for a wide range of work zone roads that can be provided by widely used mapping services like Google maps. The integration between the real-time data and the traffic simulation software, that enhance the choice of the alternative detour, is a challenge that needs to be covered. Also, these gaps include the application of Bridge Information Modeling (BrIM) in visualizing the Transportation Management Plan (TMP) used to manage the work zone traffic during the construction stages of the bridge. In addition, the gabs include a crashing process through a trade-off analysis for bridge construction items to optimize the project duration and cost, including the user costs.

## Framework components

**Figure 1:**
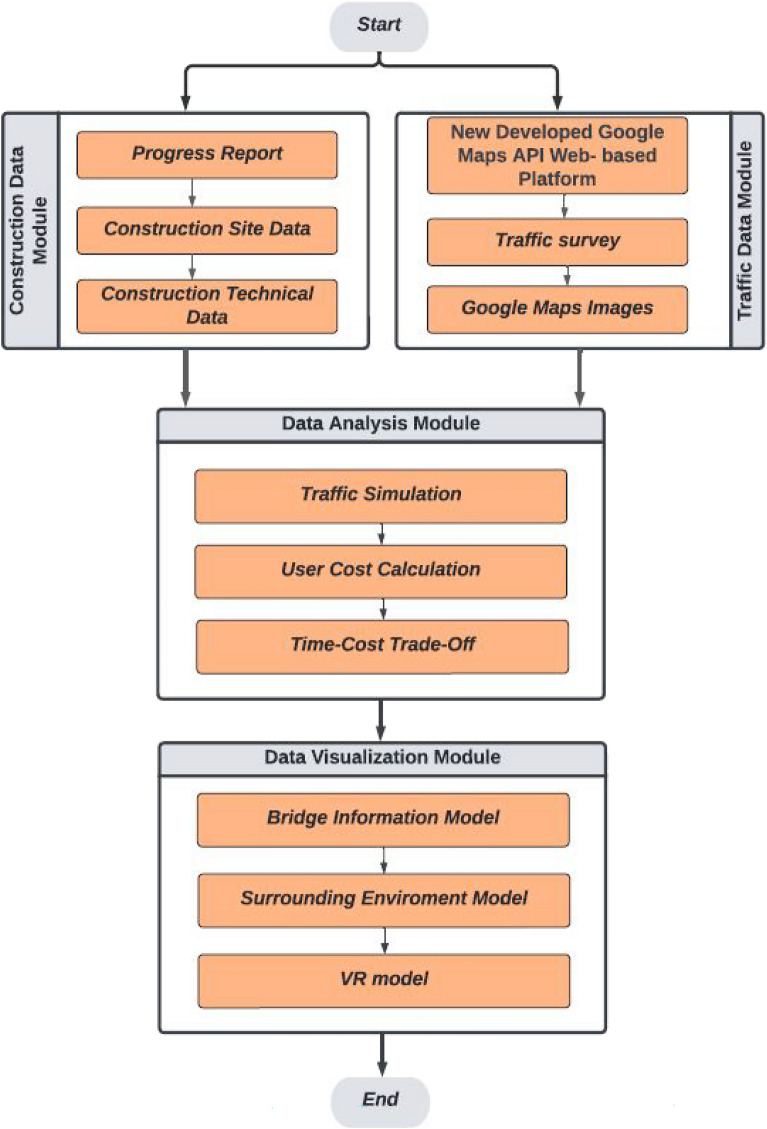
Proposed framework modules.

The proposed framework provides a methodology to manage the work zone activities of bridge construction easily and efficiently. The framework consists of four main modules, as shown in Fig. 1. The framework starts with collecting the data of the two scopes—Construction and traffic—through different methods illustrated in the two modules. The framework then applies the data analysis module by developing a traffic simulation of the work zone roads, calculating the user costs for these roads, and applying the time–cost trade-off analysis. The process is then visualized through the last module, where the scenarios of expected detours and the construction stages are visualized. The framework modules are the construction data module, Traffic data module, Data analysis module, and data visualization module.

### Construction data module

There are two types of data used in the proposed framework. The construction data is the first type of this data. It is gathered in three ways: project daily progress reports, recording construction site data, and technical documents. Firstly, the daily report contains a simple representation of the project elements in the Elevation view, Plan view, and section views. The bridge elements in these views are colored to distinguish their status (yet to start, in progress, finished, delayed, reasons for the delay, and types of obstacles).

Secondly, recording the construction site is necessary to help complete the missing information and details that are hard to illustrate in the daily progress reports. For example, the information gained from the site is the location of heavy machines, the maneuvers between the other positions in the work zone, durations to finish the required activities, and construction obstacles like infrastructure pipelines and electric towers. The site data can also help track work zone traffic status changes, like speed and volume, as the work zone occupies street lanes to serve the stored materials and equipment. These occupied lanes cause traffic congestion during rush hours and obligate travelers to use the detours if needed in case of complete closure of road lanes.

Thirdly, the project technical data in the construction stage includes the contract, the design drawing, the shop drawing, the bill of quantities (BOQ), technical project specification, the minutes of meeting (MOM), request for information (RFI), work inspection request (WIR), material receiving the request (MRR), material inspection request (MIR), site instruction form, non-conformity request (NCR). These documents represent the technical information needed in the construction stage and, consequently, needed in the proposed framework modules.

### Traffic data module

The traffic data is considered the second data source for the proposed framework, which focuses on the measurements taken from the work zone of the bridge construction site. These measurements are taken in three ways: Google Maps API, Traffic Survey, and Google Maps Images.

#### Google maps API web-based platform

The proposed framework uses Google Maps services^27^ as one of the sources of traffic data. Google Maps API is used in the proposed framework to link the services offered by Google maps with a newly developed web-based platform which allows choosing work zone roads that will be taken into consideration by defining the pass of the road from its points. The hierarchy of the web-based platform consists of three main tabs: Road, configuration, and permission.

**Figure 2:**
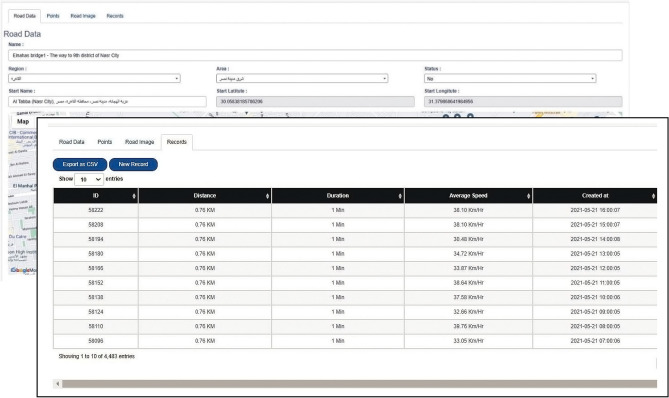
Interfaces of developed Google maps API web-based platform.

The “Roads” tab interface consists of four web pages, as shown in Fig. 2: Road Data, Points, Road Images, and Records. The first page, “Road Data” is used to insert the data related to the chosen roads. It consists of three main parts. The first part is to input the road name, region, area, and status. The second part is choosing the start position of the road from a map window and the end position from another window, where the platform will automatically determine each selected point's latitude and longitude values. The third part includes setting the start and the finish time, which determines the time range for recording data during the day. Also, it includes setting the periodic time that determines the period between each recording.

The second web page of the “Road” tab is the “Points.” It includes adding the intermediate points between the start and end points. These extra points are needed to determine the path considered in the study since Google Maps may consider other paths that give lower travel time between the start and the end points.

The third web page of the “Roads” tab is the “Road image.” It is used to view the final path of the studied road. The fourth web page is “Records,” which is used to view the recorded data in tables. The available traffic data that can be extracted from Google maps are the date and the time at which the record is created, the distance of the chosen road, the speed and the travel time of road users, and finally, the image of the chosen path. The image recorded for each data is to check the path chosen. Besides, recorded data can be exported to excel sheets using the “Export as CSV” tab for more calculations in the next framework modules.

Choosing the work zone roads where their traffic data will be recorded is performed in several steps. Firstly, all roads directly related to the work zone—main roads and surrounding roads—are studied to choose the roads affected mainly by the construction activities. This can be done by using the web-based platform to record the travel time readings for different roads surrounding the work zone before and during the first stages of construction. Then, these roads' start and end points are gradually chosen one after the other outside the work zone without any intermediate points to cover the choices Google Maps offers for the road users to get out of the expected congestion. These records show the most affected roads by construction activities and the expected detours by Google Maps, which can be permanently chosen as the source of traffic data.

Secondly, the changes happening in the traffic status of google maps for the work zone will help determine the roads that are expected to be affected the most due to the construction activities of the bridge. It distinguishes the traffic status of the roads with four colors (green, orange, red, and dark red), where dark red represents heavy traffic. Also, it can differentiate the different hours of the day and the days of the week. Thirdly, the gathered construction site data reveals the actual detours chosen by the passengers, which must be added to the list of roads where traffic data are recorded.

#### Traffic survey and Google earth images

The traffic survey in the bridge construction work zone is the second source of traffic data in the proposed framework. It is used to complete the missed data that cannot be extracted from the Google maps web-based platform, which is the traffic volume. In addition, exploring the work zone traffic status helps determine the critical roads that should be tracked and the positions that should be the surveying points.

**Figure 3:**
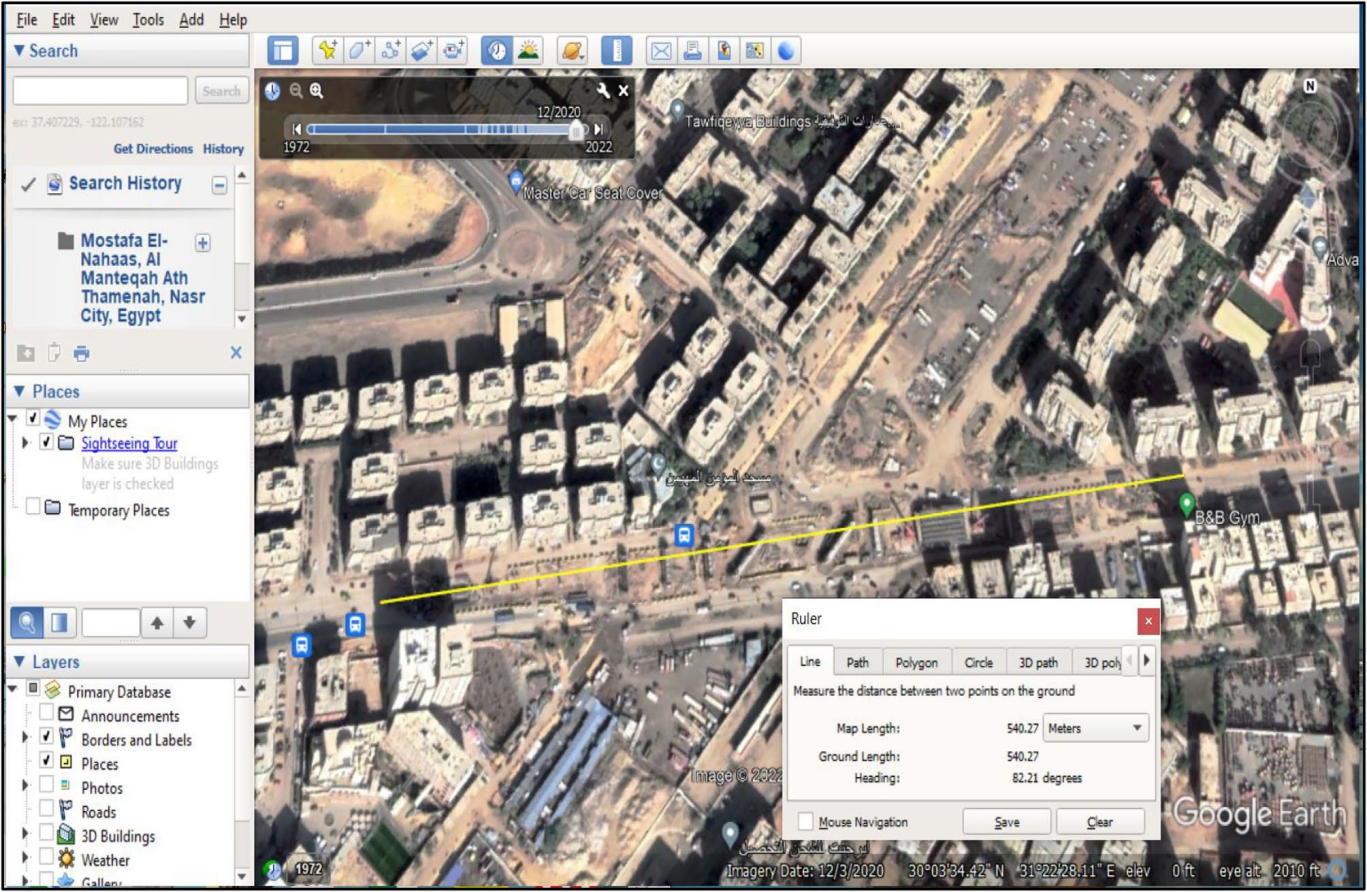
Demonstrating Work zone length and construction progress in Google Earth Images.

The third source of traffic data is Google Earth Images. It is the images produced from Google Earth after determining the location of the work zone, as in Fig. 3. Some measurements can be taken from Google Earth Images, like the queue’s lengths and the number of vehicles in these queues. Also, the authentic images of the passenger cars in the different stages can be observed using these images, which will help determine the detours road users take to get away from the congestion. On the other hand, the progress of the construction activities, the positions of cranes, and the maneuvering of site machines can be detected from these images. It is worth mentioning that the available Google Earth images cannot replace the site visits as they are captured at certain moments, while the site data allow more detailed images at the needed time.

### Data analysis module

#### User cost calculations

The user costs are the costs incurred by the users of the roads due to the deteriorated conditions of the roads, such as the narrow width, closed lanes, and temporary detours. The user cost of a particular road is the sum of the traffic delay, vehicle operation, accident, and failure costs. The accident and failure costs are out of the scope of the proposed framework. The bridge user cost can be calculated using Eqs. (1)–(3) Safi^28^.

1 $$Bridge \;User \;Cost = TDC + VOC + AC + FC$$

Firstly, the Traffic Delay Cost (TDC) results from the travel time increase in the work zone due to speed reduction, congestion delays, or increased distance due to a detour c. It can be calculated using Eq. (2):

2 $$TDC = \mathop \sum \limits_{t = 0}^{{T_{E} { }}} T*ADT_{t} *N_{t} *\left( {r_{T} w_{T} + \left( {1 - r_{T} } \right) w_{P} } \right) \frac{1}{{\left( {1 + r} \right)^{t} }}$$

Secondly, the vehicle operation cost (VOC) is the cost incurred by the road users due to operating the vehicle for additional time due to the traffic disturbance of the work zone activities and the detour. Equation (3) calculates the VOC, where the factors of the user cost equations. The description of the parameters that are used in user cost equations are listed in Table 1.

3 $$VOC = \mathop \sum \limits_{t = 0}^{{T_{E} { }}} T*ADT_{t} *N_{t} *\left( {r_{T} O_{T} + \left( {1 - r_{T} } \right) O_{P} } \right) \frac{1}{{\left( {1 + r} \right)^{t} }}$$

**Table 1:** Description of user cost equations estimation equation.

Factor	Description	Units
TDC	Traffic delay cost	$/Day
VOC	Vehicle operation cost	$/Day
AC	Accident cost	$/Day
FC	Failure cost	$/Day
L	Effected length of the bridge	Km
V_o_	Normal velocity	Km/h
V_wz_	Work zone velocity	Km/h
T_o_	Normal travel time	H
T_wz_	Work zone travel time	H
T	delay in travel time = T_wz_ − To	h
ADT	The volume of traffic passing a point or segment of a road in both directions per day	Veh./day
N_t_	Number of days needed to perform the work	Day
W_p_	Hourly time value for one passenger car	$/h
W_T_	Hourly time value for one truck	$/h
r_T_	Percentage of truck	0
r	Discount rate (r) which turn all the values to the present values to be able for usage together	0
O_p_	Average hourly operating cost for one passenger car	$/h
O_T_	Average hourly operating cost for one truck	$/h

Some assumptions are taken into consideration during the calculations of the user cost in this research; Normal traffic speed (V_o_) is measured from the field. ADT is the average daily traffic volume measured in each stage of construction. ADT is divided equally between the main road and the corresponding detour in the second stage. The Work zone speed (V_wz_) is calculated from the corresponding speed of the Peak hours at each stage. The proposed framework classifies the construction stages of the bridge into four stages based on the occupied road lanes into the following stages; Stage (1): Includes the execution of piles, pile caps, and piers. Stage (2): Includes the execution of pier heads. Stage (3): Includes the execution of beam erection and slabs. Stage (4): Includes the execution of newgersy and finishing.

#### Traffic Simulation

**Figure 4:**
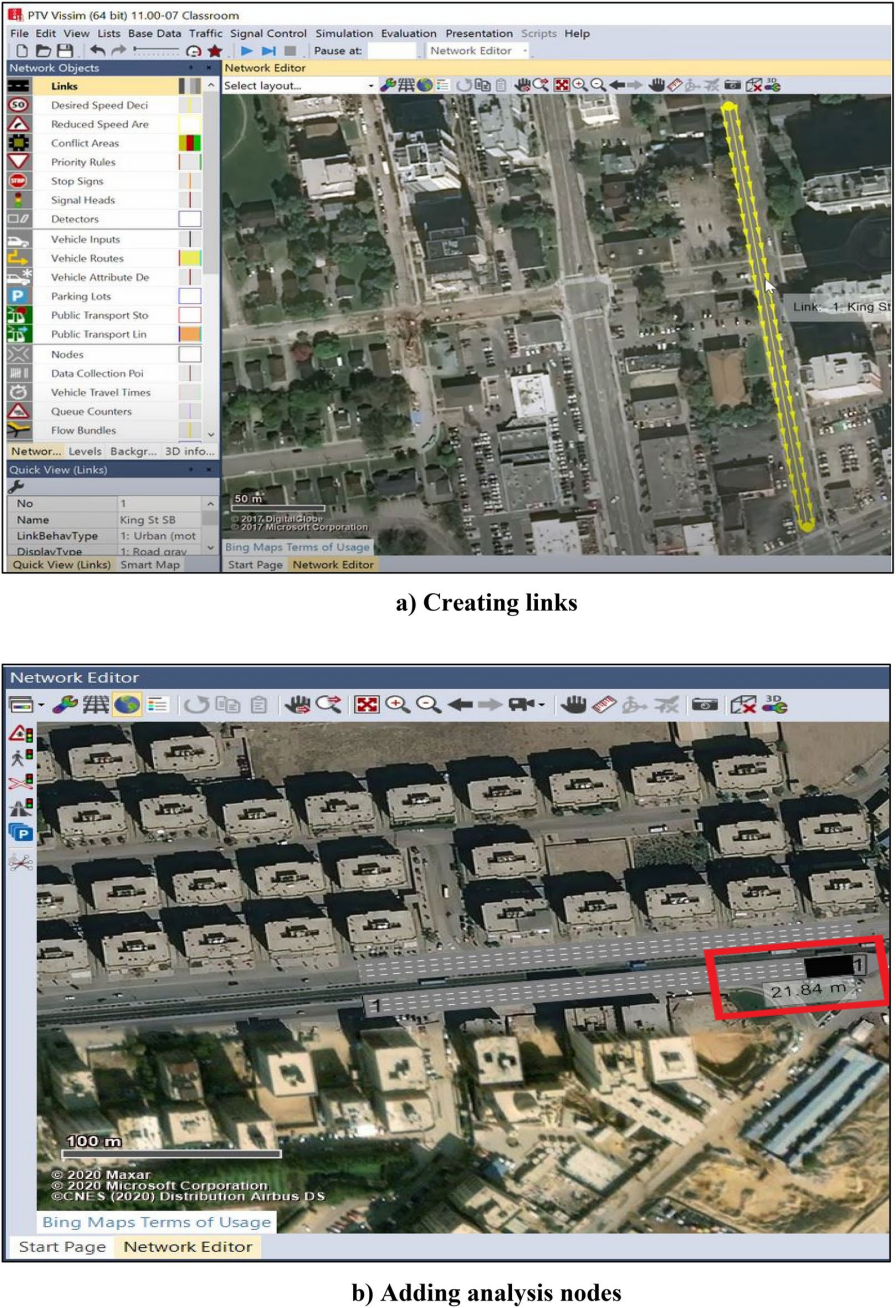
Defining VISSIM inputs for simulating bridge construction.

The software used in the traffic simulation is VISSIM^29^ which simulates one construction stage at a time. The input of the simulation process includes many steps. First, the project's location must be determined. Secondly, links must be drawn for the roads of the work zone area that will be taken into consideration during the simulation process, as shown in Fig. 4a. Finally, the characteristics of the drawn links should be determined in the link properties window. It includes link properties such as link number, name, length, number of lanes, the width of each lane, link behavior type to determine the type of the road, urban or freeway, display type to represent its color, level, etc. Thirdly, the vehicle Inputs panel is used to enter the traffic volume value (vehicle/hour). Fourthly, nodes are added to the drawn links as an analysis point where the simulation results will be based on the location of these nodes (see Fig. 4b). The simulation parameters include the simulation period in seconds, the start time and date, and the simulation resolution. The simulation results are represented in graphic representation of vehicle motion and Microsoft Excel sheets that include the results of the simulation process, which are the moving delay, stop delay, and queue length for each link.

#### Time–cost trade-off analysis

The time–cost trade-off in the proposed framework includes the indirect cost, calculated from the rent of machines, salaries, cost of the gas, water, wood for the formwork, all types of assurance, medical treatment, spare parts of machines, rent of framework, and internal maintenance. While the activities crashing, the direct cost values increase as the project duration decreases, which occurs gradually through defined steps. Firstly, the actual rate of construction and the actual cost is calculated for each stage depending on the construction data gathered from the site. Then, this actual cost is broken down into its components: the costs of the materials, machines, and manhours for each item. As the quantities of these components increase gradually—referring to the method statement of each item—the rate of construction increases, the direct cost increases, and the activity duration decreases, which forms the points of the direct cost curve. The crushability of each stage (available days to be crashed) and its crashing slope (cost increase due to crashing per day) are then calculated.

For the user cost curve of the bridge construction, the user costs are calculated based on each stage's normal duration. Then, the user costs calculations are repeated gradually according to the newly calculated project durations in each step of crashing the activities. The user cost values are expected to decrease as the duration decreases. Finally, the total cost curve is the submission of the cost curves where the values of the three types of costs (direct, indirect, and user) are added at each value of the newly calculated duration.

### Data visualization module

**Figure 5:**
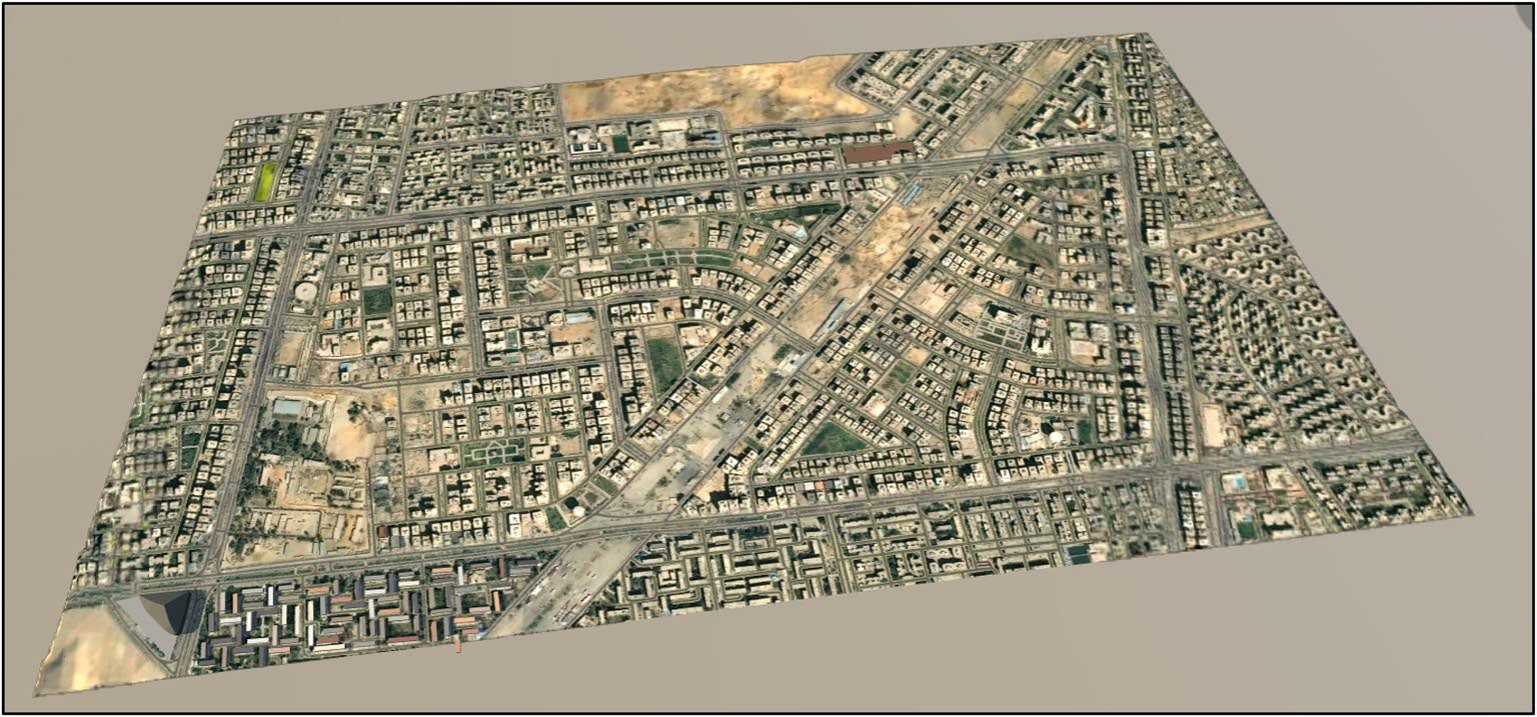
3D representation of work zone in InfraWorks.

In the proposed framework, the BrIM model is a bridge model in Autodesk Revit^30^. It contains all the required data related to the bridge construction, including the dimension of the bridge elements, the material characteristics of each element, and the cost of each unit of this element. In the proposed framework, Autodesk InfraWorks^31^ model captures the surrounding environment around the bridge work zone, as shown in Fig. 5. This environment includes streets, buildings, the topography of the natural ground, the lighting poles, gardens, and all other components that exist in real life. This environment is where the work zone exists, and the construction impact is revealed on the traffic passing through this environment.

**Figure 6:**
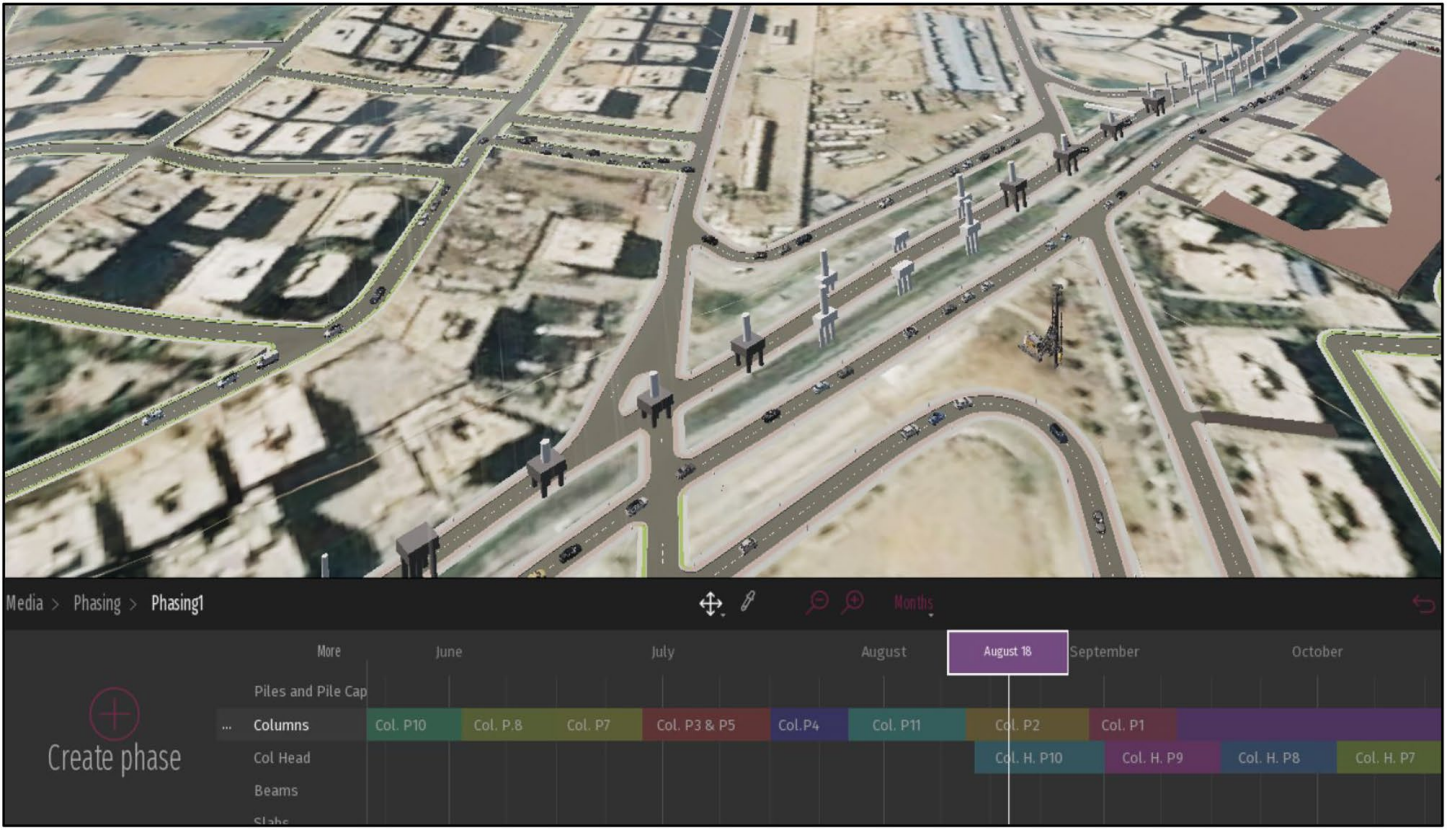
Twinmotion phasing for construction activities and traffic Representation.

Twinmotion^32^ is used to visualize the whole framework process, in many steps. Firstly, the (.fbx) file of the bridge BIM model and the (.fbx) file of the InfraWorks model are required to be imported into Twinmotion. Secondly, the bridge BIM model is placed and oriented concerning the surrounding environment. Thirdly, the elements existing in reality, such as cars and construction equipment, may be added to the model. The added elements, like cars, represent the traffic status of the work zone. These elements are assigned to specific pathways that road users in the work zone already use. Fourthly, the Twinmotion model allows the visualization of the different construction stages and their traffic impact through a specific property called (Phasing) as depicted in Fig. 6. The phasing property consists of tracks where each track consists of tasks. The tracks represent different construction stages defined in the framework, while the tasks are the bridge elements in each stage. Twinmotion allows the creation of many phasing cases representing the scenarios expected to happen in the framework. Fifthly, the model of Twinmotion allows the user to create videos about the expected scenarios of work zone construction activities.

## Case study

**Figure 7:**
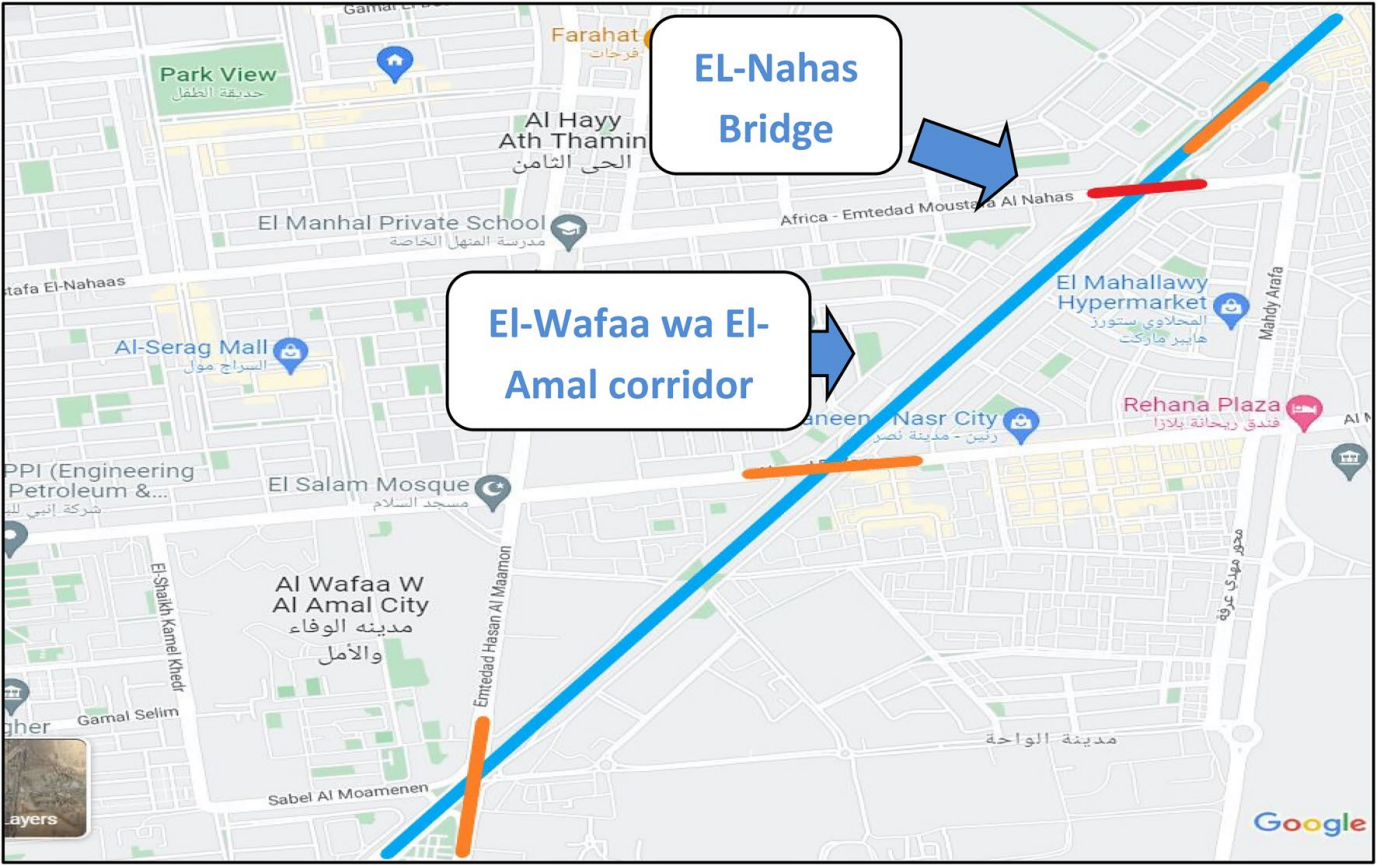
The Intersection of El-Wafaa wa EL-Amal corridor and EL-Nahas bridge.

The case study is located in Naser city, Cairo, Egypt. It is a part of the construction of a corridor that links Naser city with ElSuez highway, as depicted in Fig. 7. The construction of the corridor includes the construction of the case study bridge (Mostafa El-Nahas bridge), located at the intersection between Mostafa El-Nahas Street and the corridor of El-Wafaa wa El-Amal (Shinzo Api corridor).

### Construction data collection

The data collected for the construction stage of the case study include the same components of the construction data module. The report contains the components of the bridge, where each color represents the current status of the component. It also contains tables that capture the current status of these components with additional data about the available items, the total number of items, and the percentage of progress. The second step in the construction data module is gathering site information (e.g., cranes, concrete pumps, etc.) and recording the updates and changes. The cranes and the concrete pump positions were recorded according to the served element. For the erection of beams, very heavy cranes are used for lifting the beams that may weigh up to 100 tons, and specialized trucks may be elongated to 30 m in length.

**Figure 8:**
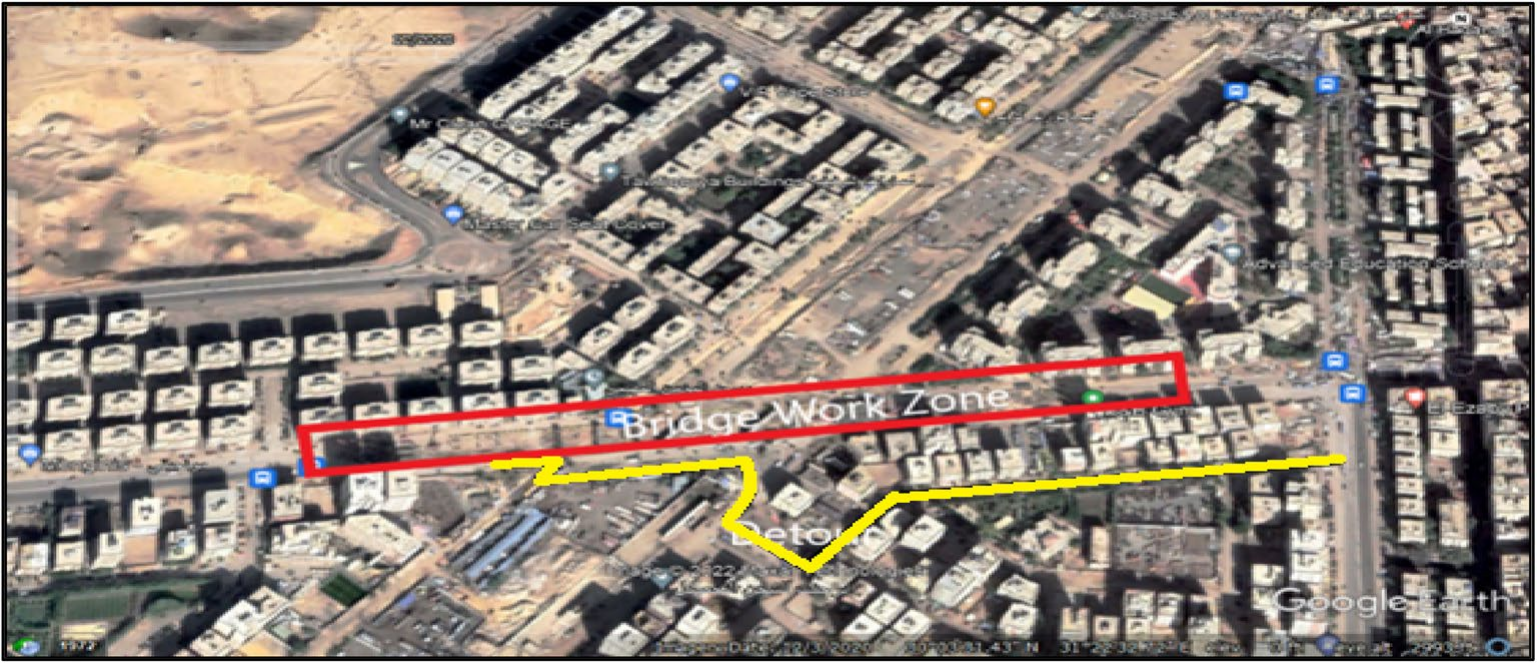
Actual traffic detours for the case study.

In addition, the detours are detected gradually as the road users discover new roads to overcome the congestion of the work zone. The main detours that the whole traffic was obligated to use were in the stage of beam erection, as in Fig. 8, where all the main road lanes were entirely closed by the heavy cranes and the precast beam trucks.

The construction activities' obstacles were also detected in the site data recorded. In the case study, the elevated electric cables and electrical towers crossing the bridge path represent are the construction obstacles representing danger in the work zone. Also, underground utilities, like water pipelines, sewage, gas, and telephone lines underneath the work zone ground, should be considered during construction work, especially for the excavation and pile digging activities. The available technical data is represented in the form of the BOQ of the project, the project specification, the design drawing, and the shop drawing. This data is used to develop the bridge's BIM modeling and complete the user cost calculations.

### Traffic data collection

**Figure 9:**
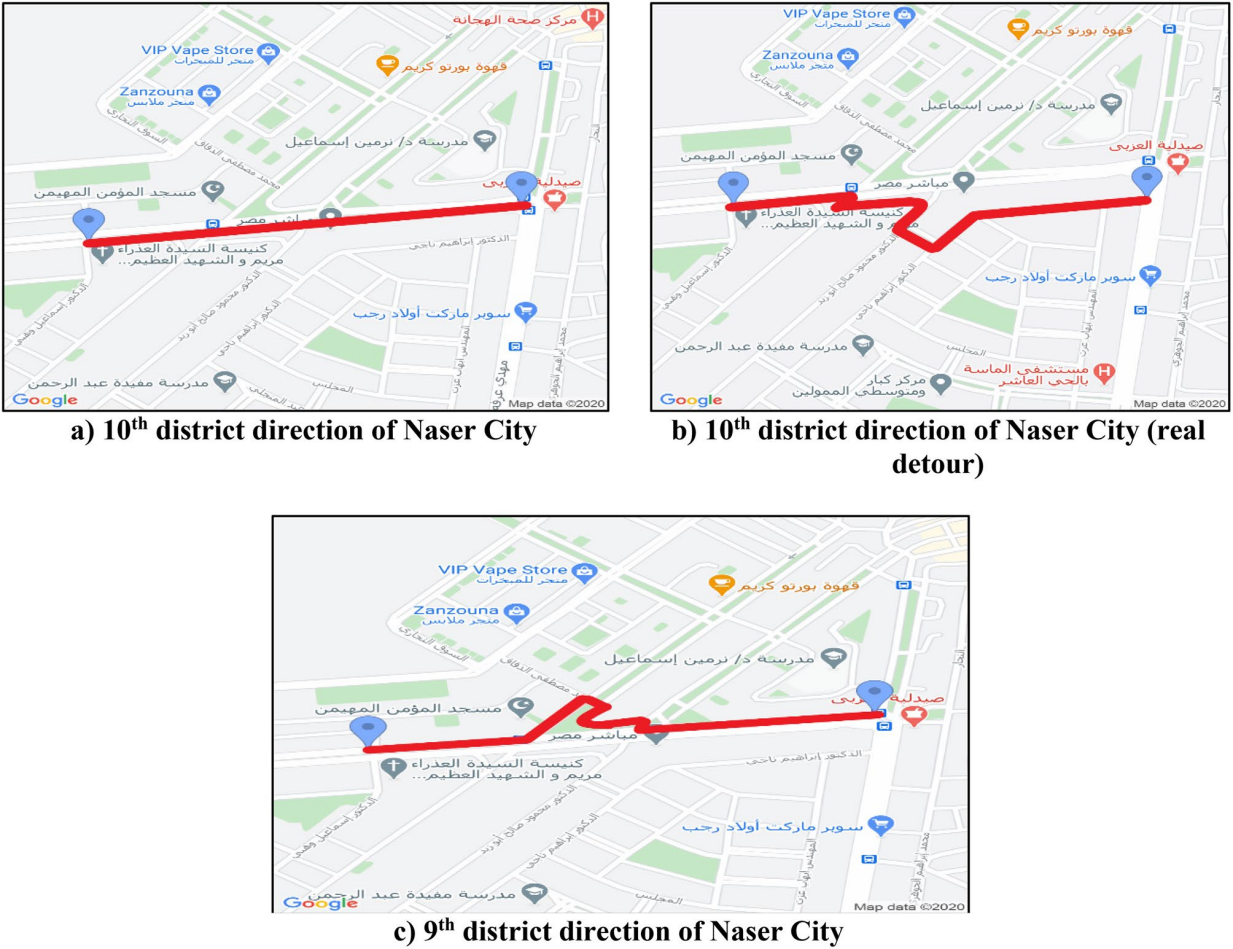
Roads recorded data for Mostafa El-Nahas street.

For applying the Google Maps API in the case study, a web-based platform is created and linked with services offered by Google Maps to use its API. In order to explore the traffic status of the work zone, 15 roads were selected to be monitored, and their data was recorded. The data recorded from these roads are used to determine the significant roads affecting the work zone. The roads chosen to be monitored throughout the project, as shown in Figure 9, are the 10th district direction of Naser City and the 9th district direction of Naser City.

**Figure 10:**
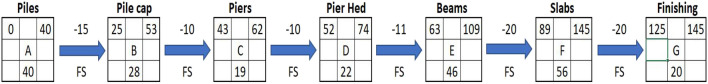
Diagram for durations and lags of bridge activities.

The activities of the bridge construction, as shown in Figure 10, are divided according to the occupies lanes in the work zone. Accordingly, the data is divided into specific dates according to the stages of construction, as listed in Table 2. The maximum, minimum, and average values of the recorded data are illustrated in Table 3.

**Table 2:** Case study construction stages.

Stages	Stages items	Start date	End date
1	Piles, pile cap, pier	19/10/2021	19/12/2021
2	Pier head	9/12/2021	30/12/2021
3	Beams erection and slabs	20/12/2021	11/3/2022
4	Newgersy and finishing	20/2/2022	11/3/2022

**Table 3:** Travel time values of road (10th district direction).

Stages	Road	Minimum (h)	Maximum (h)	Average (h)
1	Main	0.03	0.11	0.0528
2	Main	0.03	0.15	0.069
2	Detour	0.05	0.13	0.077
3	Detour	0.05	0.13	0.074
4	Main	0.03	0.09	0.0528
4	Detour	0.06	0.08	0.0676

### Traffic survey

The traffic survey in our case study was done on the chosen roads for monitoring to complete the data needed for the analysis. As depicted in Table 4, the survey records were recorded in forms prepared for different types of vehicles. Moreover, the recording days were chosen randomly to neglect events like feasts and holidays. Subsequently, the survey records are filtered and cleaned to delete the unreasonable records and outliers that may be human errors. Next, records are divided into the construction stages, as illustrated in Table 2, to get the records of each stage. Finally, the average of each stage record is calculated for the traffic simulation and the user cost calculations, which are used in the Time-Cost trade-off for the whole process. The values of these average records are listed in Table 5.

**Table 4:** Traffic survey records during the construction stage.

No.	Date	Time	To 10th district (Veh./h)	To 9th district (Veh./h)
1	9–12–2021	3:30 pm	1098	864
2	22–12–2021	6:40 pm	1440	840
3	28–12–2021	4:30 pm	1071	672
4	13–1–2022	6:15 pm	771	1260
5	18–1–2022	5:00 pm	1512	1128
6	26–1–2022	7:00 pm	1104	1040
7	1–2–2022	5:25 pm	1080	––
8	10–2–2022	4:40 pm	1068	1680
9	16–2–2022	6:18 pm	1332	1050
10	24–2–2022	9:00 pm	1164	1224
11	1–3–2022	7:30 pm	1956	1000
12	9–3–2022	8:10 pm	1092	1500
13	15–3–2022	7:16 pm	1848	1380

**Table 5:** The average traffic volume during each construction stage.

Stages	Stages scope	Average traffic volume (veh./day)
1	Piles, pile cap, pier	43,490
2	Pier head	39,140
3	Beams erection and slabs	28,440
4	Newgersy and finishing	32,708

### Data analysis

The Added User Cost for the chosen roads was calculated using the Safi^28^ equations. These calculations are repeated in each stage of the four-construction stage for the chosen roads. The calculations are done in Microsoft Excel sheets, as in Table 6. The values of the time delay cost and the vehicle operation cost used in the calculations are 25 LE/Day and 0.175 LE/Day, respectively. These values are adopted by Gabr et al.^33^ after making necessary adjustment to capture inflation.

**Table 6:** User cost calculation for the four construction stages.

Parameter	Stage 1	Stage 2		Stage 3	Stage 4	
	Main road	Main road	Detour	Detour	Main road	Detour
L (m)	540	540	970	970	540	970
T_o_ (h)	0.009	0.009	0.0161	0.0161	0.009	0.0161
T_wz_ (h)	0.0528	0.069	0.077	0.074	0.053	0.068
T (h)	0.0438	0.06	0.068	0.065	0.0438	0.0587
ADT (veh./day)	43,490	19,570	19,570	28,440	16,354	16,354
TDC	47,622	29,355	33,270	46,216	17,908	23,997
VOC	333	206	232	324	125	167
Bridge user cost (LE)	47,955	63,063		46,540	42,197	

For all stages V_o_ = 60 V_o_ Km/h; N_t_ = 1; W_p_ = W_T_ = 25; O_p_ = O_T_ = 0.175.

**Figure 11:**
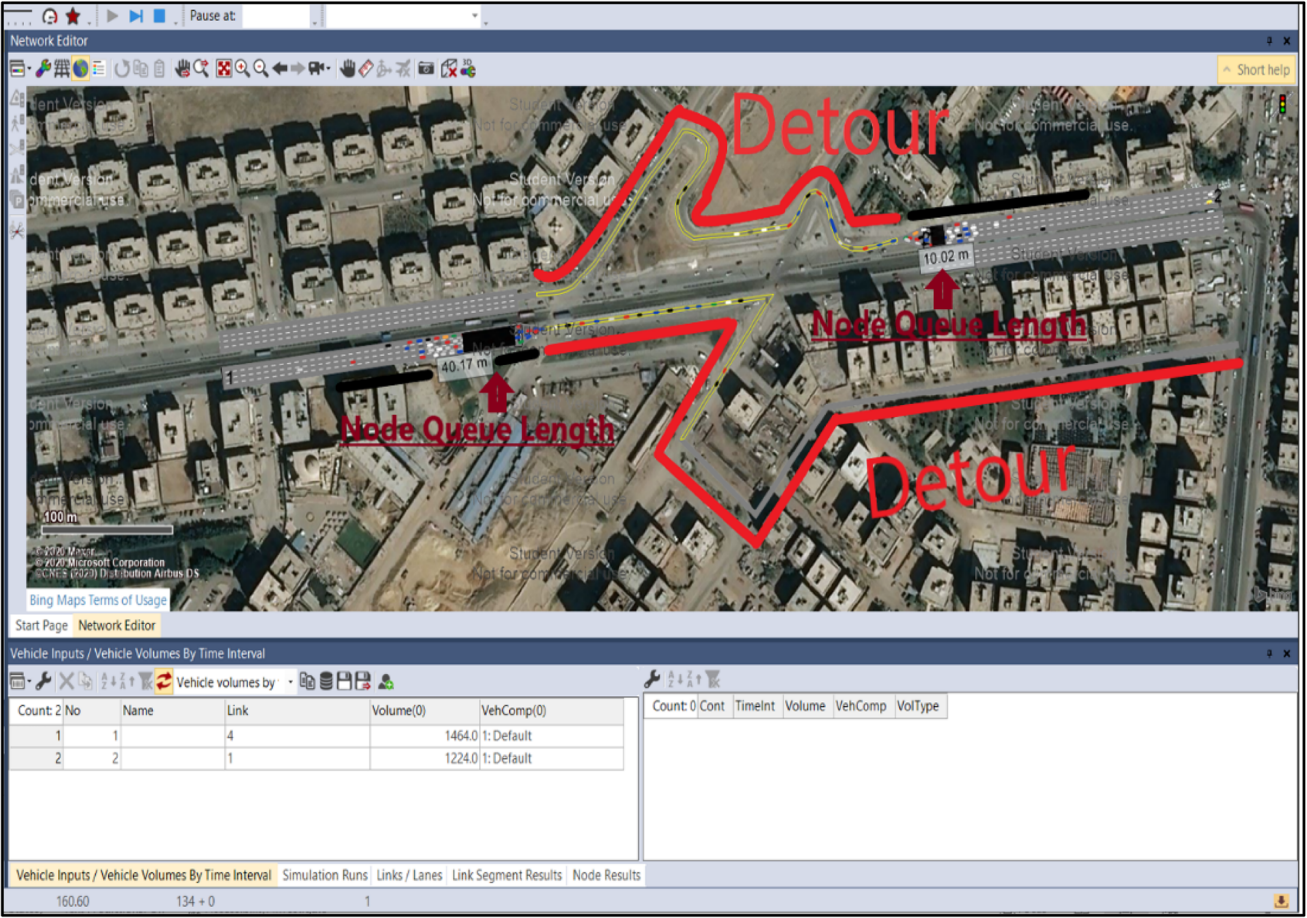
VISSIM interface showing queue formation and the detour usage.

Building a simple simulation model for the case study, as in Fig. 11, starts by capturing the location of the work zone of the bridge. In the case study, the location is the intersection of Mostafa El-Nahas Street and El-Wafaa w El-Amaal bridge in Naser City. The number of lanes is four on the main road before the work zone and only one on the main road of the work zone and detour. The volume assigned to the main road to the tenth district (Road 1) is 1464 vehicles/hr. The volume of the main road to the 9th district (Road 2) is 1224 vehicles/hr. The speed assigned to the links is 5 km/h. The node analysis is the type of analysis used in the model. Two nodes are placed in the model where the first node is placed before the work zone to the 10th district, whereas the second node is placed after the work zone to the 9th district.

### Time–cost trade-off

The time–cost trade-off is applied by calculating the daily indirect, direct, and user costs during the crashing process. First, the average daily indirect cost is calculated by adding all the sources of indirect costs and dividing it by the total number of project days. As shown in Table 7, the direct cost is calculated after crashing the activities and calculating the increased costs in each step of the crashing process. The cost breakdown does this for the bridge item. In the piling stage, the recorded situation from the site is the usage of 2 pile rigs in the stage of pile construction. This can be crashed by using an additional rig only since adding more rigs will be unreasonable for this type of bride and for maneuvering the machines. For the stage of beam erection, the crashing occurred by adding two more cranes to start the erection process in parallel with the two existing cranes. The other stages of crashing depend on increasing the formwork and the material used to achieve the crashing of the activity, as listed in Table 8. The indirect cost is calculated by recording the indirect cost of six months, which is 21,895,127 LE.

**Table 7:** Direct cost and duration of activity normal and crashing conditions.

Activity	Direct cost (LE)		Duration (days)	
	Normal	Crash	Normal	Crash
Piles	15,028,983	19,708,806	40	30
Piles caps	9,221,826	10,069,179	28	20
Piers	6,954,401	7,217,743	19	13
Pier Head	11,645,700	12,596,987	22	17
Beams	27,582,486	27,941,508	46	35
Slabs	20,757,171	22,938,861	56	44
Newgersy and finishing	4,385,353	4,489,881	20	15
Total	95,575,920	104,962,965	145	88

**Table 8:** Allocated resources in normal and crash conditions.

Stage	Normal condition	Crash condition
Piles	2 Rigs	3 Rigs
Pile cap	4 Formwork	8 Formwork
Pier	2 Formwork	3 Formwork
Pier head	4 Formwork	6 Formwork
Beam erection	2 Cranes	4 Cranes
Slab	2 Formwork	4 Formwork
Newgersy and finishing	2 Formwork	4 Formwork

### Data visualization

**Figure 12:**
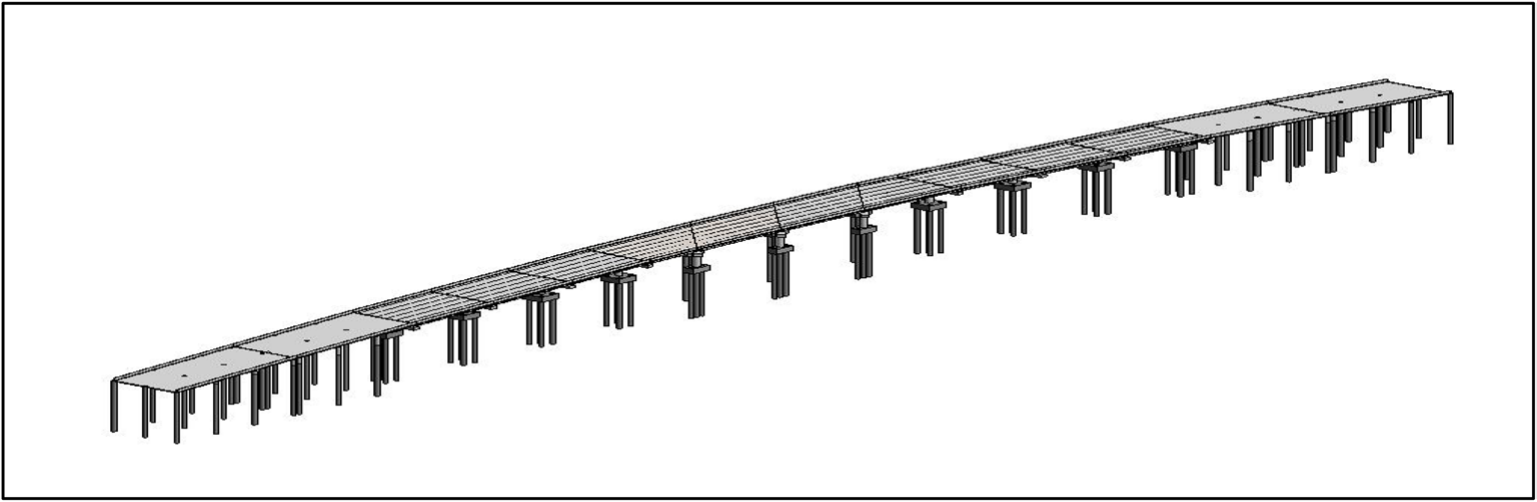
BrIM model of El-Nahas bridge.

**Figure 13:**
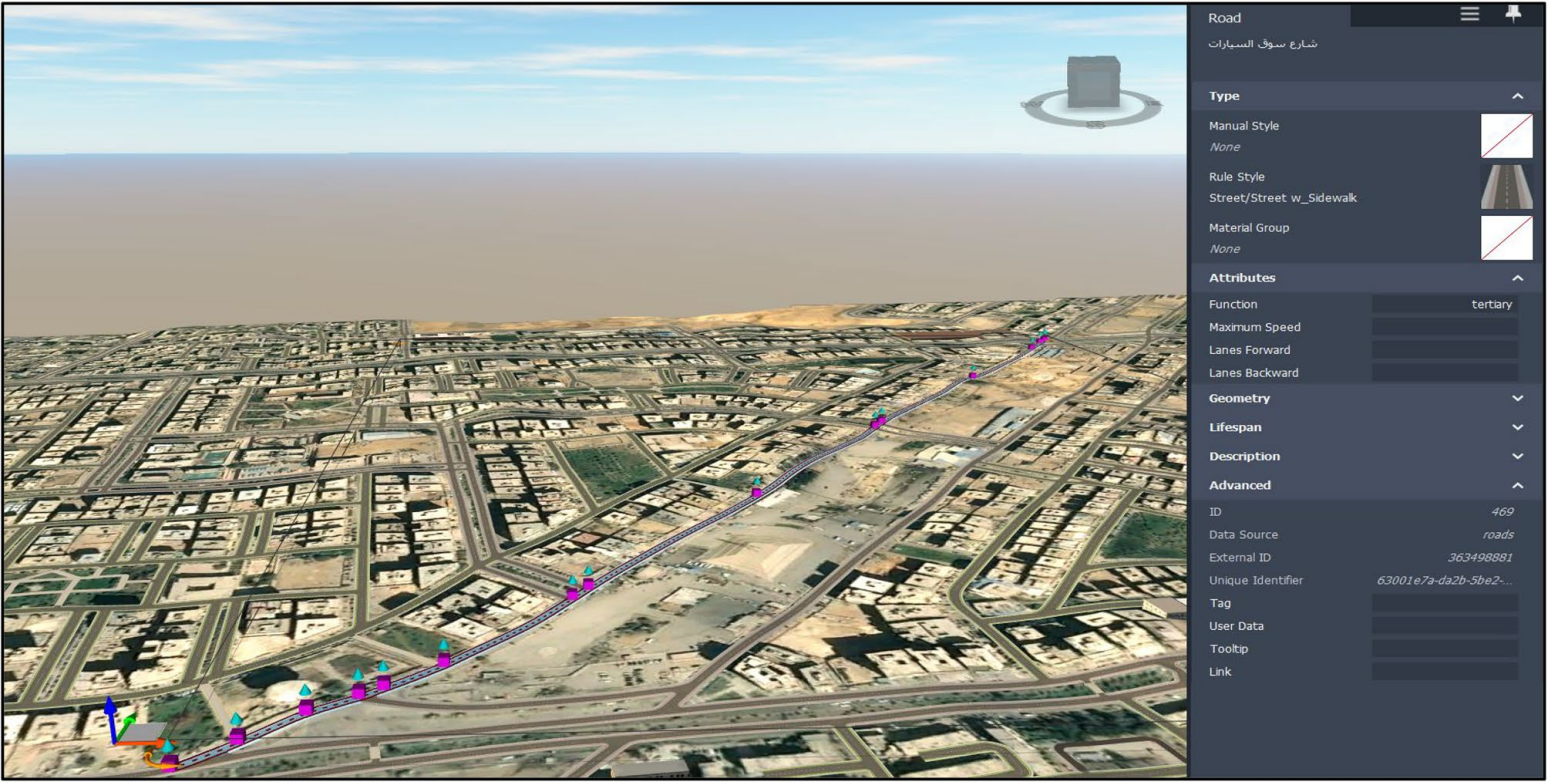
Modelling of the surrounding environment.

The first step in the visualization process is to build the BIM model as in Fig. 12 for the bridge, called the Bridge Information model. The total dimensions of the bridge elements and the detailed materials information are used to form the BrIM model of the bridge. The model can mainly read the environment properties where they are exported as standalone elements in the following steps when the model is exported to another platform. The model of Autodesk InfraWorks, as shown in Fig. 13, is used as a part of the visualization process to represent the surrounding environment where the project's work zone is part of it. The model captures the natural elements of the environment like roads, intersections, bridges, buildings, parks, and any existing natural forms like rivers, mountains, farms, etc. The model can also identify and select some aspects of the environment and read their properties, which are exported as standalone elements in the following steps when the model is exported to another platform.

**Figure 14:**
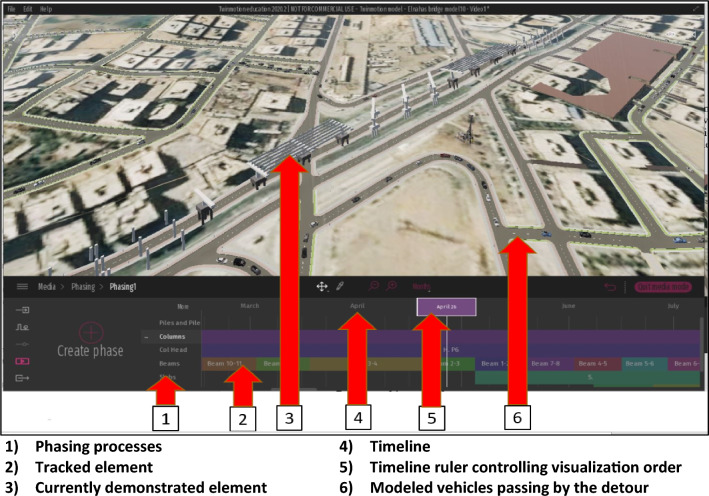
Visualizing the process by Twinmotion.

The model of Twinmotion, as shown in Fig. 14, gathered the different models and data on the same platform. It receives the exported Bridge model from Revit and the InfraWorks model file, then by using the abilities of Twinmotion—3D models for cars are created to take the path of the main road and detours of the work zone that were revealed in the gathered site data. After that, the stage's phasing occurs by classifying the bridge into its main elements and initializing a track for the main element. For each track, each element is created in its corresponding track, named by its axis, and located in its specific date of construction determined by the timeline above the tracks. The paths passengers take throughout the work zone, whether on the main road or the detours, are also called tracks. After filling the tracks with the bridge elements, the paths of the cars are located corresponding to the elements constructed simultaneously to be visual. In contrast, the element appears when the time liner crosses over them. A video of this phasing process is then created, which can illustrate the whole process of the bridge construction and the impact on the work zone traffic status from the project start to its end.

## Results and discussion

### Traffic simulation results

**Figure 15:**
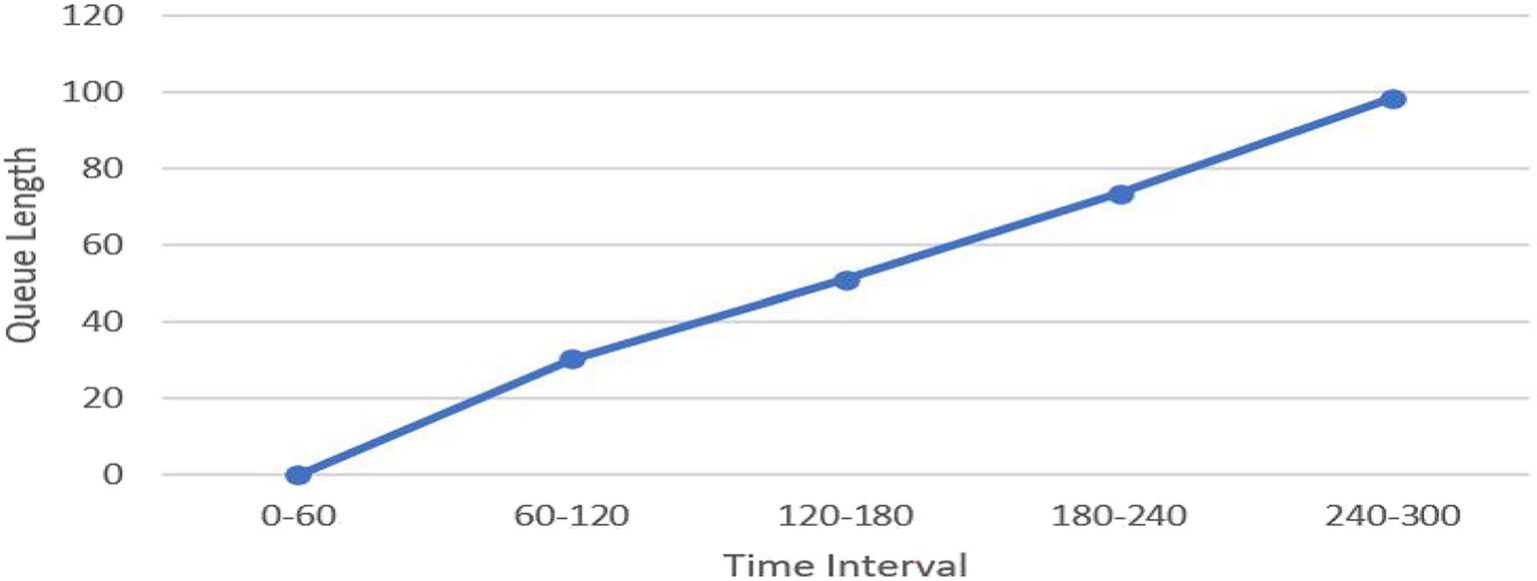
The Node analysis results in a graph for the 10th district Direction.

The node analysis results, as shown in Fig. 15, are exported to Microsoft Excel sheets containing the values of the parameters measured during the simulation. The simulation duration lasted 300 s and the results were recorded every 60 s. The results showed that:

1. No queue length is formed (no congestion) during the first 60 s; however, the average vehicle delay readings count. This is due to the reduced speed, as vehicles slow down while getting closer to the work zone. The delay here concerns the theoretical travel time, which occurs when no other vehicles or no signal controls or other reasons for stops. The non-formation of the queue represents the early morning hours or the last hours in the day before the occurrence of rush hours in reality. It also may represent in reality the early beginning of the project where the mobilization stage had no significant effect on the road. In the second stage, the queue starts to be formed because of interaction and maneuvering occurs between the construction machines and the passenger cars. Activities such as unloading the trucks, storing the concrete mixers near the bored piles, transferring the formwork between the bridge axes increase the queue length of passenger cars. As construction activities increased, the occupied lanes and the moving machines increased which cause more congestions and longer queue length accordingly.
2. The maximum queue length reached within 300 s simulation duration is 98.42 m. Any proposed detours in this distance will be unfunctional due to the formed queue length if the studied traffic conditions reach or exceed the simulation circumstances.
3. The rate of queue formation, which is 0.328 m/s, determines the available detour depending on the duration of the congestion and the corresponding traffic volume.
4. In the case of different projects, the traffic simulation is expected to give different results due to changing the simulation conditions and the different nature of each project and work zone.

### Time–cost trade-off results

**Figure 16:**
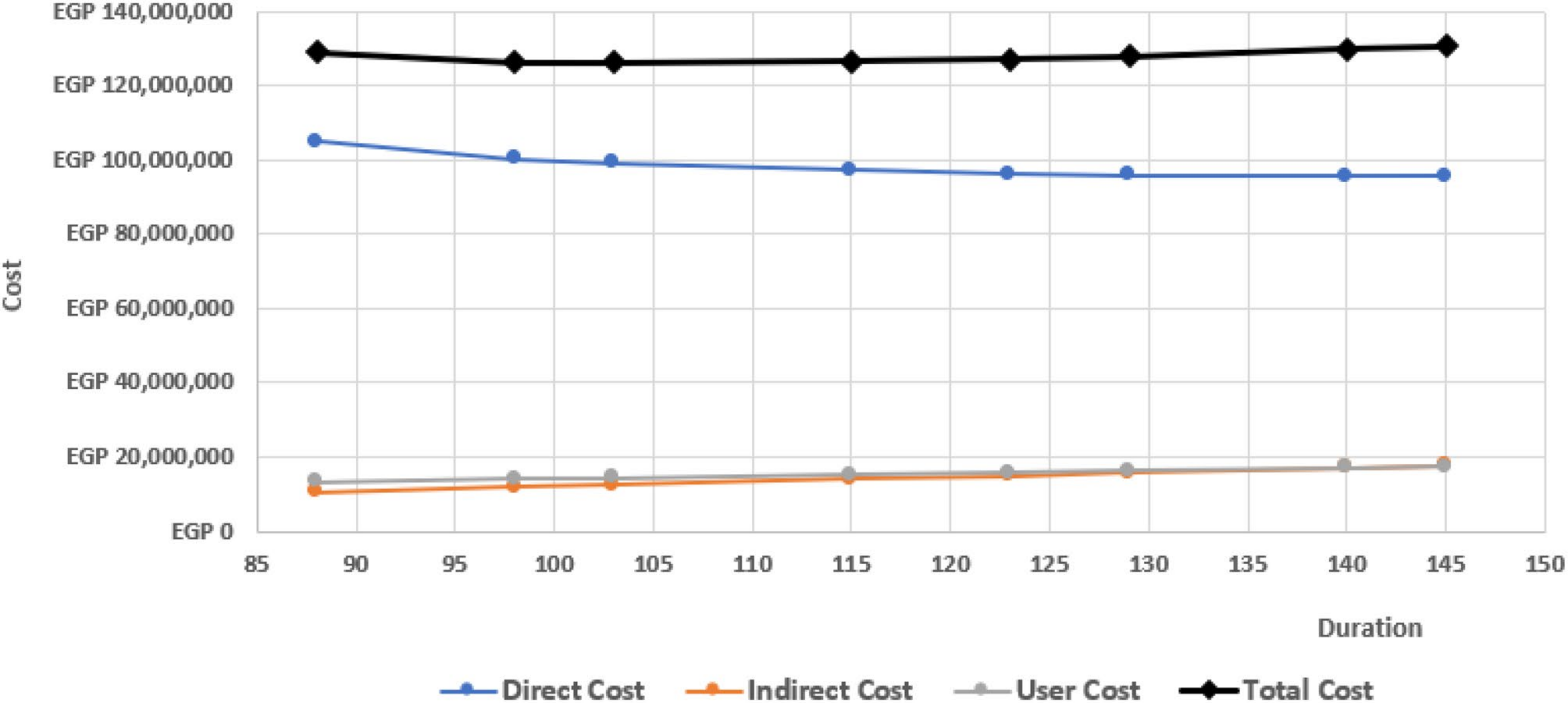
Estimated costs for Normal and Crashed Scenarios.

Table 9 lists the different effects of the construction stages on the traffic status represented in the values of the user cost. These costs start with a significant value at the first stage of construction, which are piles, pile caps, and piers which reflect the congestion that happened at the beginning of the project due to the mobilization and high transitions of the equipment and machines. Also, the road users used to take the main road in the work zone during their travel without using any detours to avoid congestion. Then, the user costs in the second stage increased due to occupying more road lanes and the rest of Stage 1 activities that continue in Stage 2. The third stage has the lowest effect on the user cost as the passenger cars still use other detours while the main road is completely closed, and the traffic is converted to the primary detour. Finally, the values in the last stages start to increase again, which means the return back of road users to use the main road. The direct, indirect, user, and total costs for the normal and different crashed scenarios as depicted in Fig. 16. The project's minimum total cost (i.e., direct, indirect, user costs) is LE 126,184,662 corresponding to a minimum total duration of 98 days.

**Table 9:** Bridge User cost values for each construction stage.

Stages	Stages activities	Bridge user cost (LE/day)
1	Piles, pile cap, pier	71,933
2	Pier head	94,595
3	Beams erection and slabs	69,810
4	Newgersy and finishing	63,296

The crashing process for the construction stages has a positive effect on the traffic flow and accordingly the user cost. Decreasing the time consumed for executing the construction activities leads to decreasing the duration of road lanes occupied by the construction machines and formwork. This will allow more traffic volume to cross the work zone which will decrease the effects of congestion caused by bridge construction represented in operation costs and the time delay cost for the road users. In order to make balance between the crashing process and the traffic flow priority, the user cost was included in the Time–Cost trade-off. This will facilitate reaching the balance.

## Conclusions

This research presented a framework for visualizing and mitigating the effect of the construction stages of bridges as one of the transportation infrastructures on the traffic of the work zone. The proposed framework includes construction data collection, traffic data collection, analysis of the collected data, and visualizing the data and the analysis results. Construction data collection is achieved by recording the engineering data, project management data, and effective incidences like equipment positions and maneuvering from the site through all the project stages. Traffic data is collected from the newly developed web-based platform that collects real-time traffic data using Google Maps API, Google earth images, and traffic surveys from the work zone roads. The analysis of the collected data includes the traffic simulation for the work zone roads to determine the queue length of the formed congestion and the available roads that can be considered alternative detours. The analysis also includes the user cost calculation for the main road and alternative detours for the different stages of bridge construction to show the effect of each stage on the work zone traffic in order to take precautions in the traffic management plan and use the suitable traffic control tools for mitigating this effect and minimize costs of road users. The analysis ends with a time–cost trade-off process for the bridge construction to optimize the relationship between the construction cost and the user cost during the project crashing. Finally, the framework visualizes the data collected and the results conducted from the analysis through BIM technology by building a BrIM model for the bridge, with the transportation infrastructure considered. Then integrating the BrIM model and the surrounding work zone environment in a visualization software capable of adding additional 3D items and project schedules to perform and study different scenarios that enhance the decision-making process in the traffic management plan (TMP). The framework was applied to a real project of bridge construction.

The case study results revealed that the second construction stage for bridges, which include the construction of piles, pile cap, piers, and pier head is the most effective stage on the work zone traffic. The research results revealed the importance of studying the alternatives for the road detours during the different construction stages using the Bridge information modelling and the traffic simulation. The simulation of work zone in the BIM and real Engines facilitate the decision making in choosing the relevant detours while the traffic simulation verify the detours using analysis factors such as the queue length values. The combination of simulation process and the time–cost trade-off optimizes the overall cost from the construction point of view and the traffic point. The proposed research aids in deciding which construction stages to be crashed and which verified detour to be chosen for the road user.

The proposed framework has a number of limitations. First, the developed web-based platform connected to the Google Maps API records the speed and the travel time of the selected roads. These records are considered to be average values for the passenger car using Google maps API, as it does not represent the travel time of all cars using the road. Also, the framework doesn’t take into consideration other activities accompanying the construction of infrastructure bridges that highly affect the work zone traffic like moving underground utilities. This research can be extended in the future to enhance the platform records by adding records measured from the work zone to be more accurate. Further, other activities accompanying the construction of infrastructure bridges that highly affect the work zone traffic, like moving underground utilities that hinder the construction of the bridges. The results of the framework can be analyzed and compared using different cases studies with different aspects (i.e., sizes, complexity, bridge type and location), performing sensitivity analysis to identify the most influential aspect on the results.

## Data Availability

The datasets generated and/or analysed during the current study are not publicly available due project data confidentiality agreement but are available from the corresponding author on reasonable request.
